# Acquirement of the autonomic nervous system modulation evaluated by heart rate variability in medaka (*Oryzias latipes*)

**DOI:** 10.1371/journal.pone.0273064

**Published:** 2022-12-30

**Authors:** Tomomi Watanabe-Asaka, Maki Niihori, Hiroki Sonobe, Kento Igarashi, Shoji Oda, Ken-ichi Iwasaki, Yoshihiko Katada, Toshikazu Yamashita, Masahiro Terada, Shoji A. Baba, Hiroshi Mitani, Chiaki Mukai

**Affiliations:** 1 Space Biomedical Research Office, JAXA, Tsukuba, Japan; 2 Department of Integrated Biosciences, Graduate School of Frontier Sciences, The University of Tokyo, Kashiwa, Japan; 3 Faculty of Medicine, Tohoku Medical and Pharmaceutical University, Sendai, Japan; 4 Department of Biology, Faculty of Science, Toho University, Funabashi, Japan; 5 Department of Social Medicine, Division of Hygiene, Nihon University School of Medicine, Tokyo, Japan; 6 ISS Science Project Office, JAXA, Tsukuba, Japan; 7 Department of Biology, Ochanomizu University, Tokyo, Japan; Karlsruhe Institute of Technology, GERMANY

## Abstract

Small teleosts have recently been established as models of human diseases. However, measuring heart rate by electrocardiography is highly invasive for small fish and not widely used. The physiological nature and function of vertebrate autonomic nervous system (ANS) modulation of the heart has traditionally been investigated in larvae, transparent but with an immature ANS, or in anesthetized adults, whose ANS activity may possibly be disturbed under anesthesia. Here, we defined the frequency characteristics of heart rate variability (HRV) modulated by the ANS from observations of heart movement in high-speed movie images and changes in ANS regulation under environmental stimulation in unanesthetized adult medaka (*Oryzias latipes*). The HRV was significantly reduced by atropine (1 mM) in the 0.25–0.65 Hz and by propranolol (100 μM) at 0.65–1.25 Hz range, suggesting that HRV in adult medaka is modulated by both the parasympathetic and sympathetic nervous systems within these frequency ranges. Such modulations of HRV by the ANS in adult medaka were remarkably suppressed under anesthesia and continuous exposure to light suppressed HRV only in the 0.25–0.65 Hz range, indicating parasympathetic withdrawal. Furthermore, pre-hatching embryos did not show HRV and the power of HRV developed as fish grew. These results strongly suggest that ANS modulation of the heart in adult medaka is frequency-dependent phenomenon, and that the impact of long-term environmental stimuli on ANS activities, in addition to development of ANS activities, can be precisely evaluated in medaka using the presented method.

## Introduction

Living organisms continually respond to various types of environmental stress. The autonomic nervous system (ANS) plays the central role in adjusting the various physiological parameters in coordination with the hormonally-regulated endocrine system in vertebrates. The vertebrate heart responds to changes in physical and physiological conditions by regulating the heartbeat and accumulated evidence supports the notion that the vertebrate heart rate is regulated by the ANS, which comprises sympathetic and parasympathetic nervous systems [1]. Both branches of the ANS regulate cardiac activity; the parasympathetic system decreases, whereas the sympathetic system increases steady-state heart rate [2]. They also modulate heart rate variability (HRV) and various types of analysis have been developed to investigate HRV modulation by the ANS and to understand how the vertebrate ANS functions in humans and also in fish [3–7]. since mammals and teleost fish share a true ganglionated sympathetic trunk together with a distinct vagal system that is similar to that in mammals [8].

The function of the ANS in cardiac regulation has been investigated using electrocardiographic HRV analysis in anesthetized adult fish, as well as heart rate analysis using video imaging in embryos or in larvae just after hatching since their body trunks are transparent [9–12]. Analysis using electrocardiography (ECG) is effective to find intrabeat abnormalities of heart, such as QT prolongation and studies using large fish such as scorpion fish or rainbow trout have been conducted using implanted electrodes [11, 13]. Analysis using ECG is also tried in small fish like zebrafish, however, the method using needle electrodes is highly invasive for small fish and it also requires that the fish was placed on its back and water with anesthesia or muscle relaxants flowed in through fish mouth by tube [7, 11, 14], so that there are serious difficulties to acquire HRV data in intact fish which is the target of this study. It is important to reduce the impact on the fish during measurement and the invasiveness of the measurements needs to be minimized. Although a popular anesthetic MS-222 (Tricaine) interferes with sympathovagal activity in fish [10, 15], anesthesia is nevertheless required to conduct the electrocardiography measurement in adult fish and amphibians [13, 16, 17].

Imaging technologies is another option to measure cardiac activity and less invasive than electrocardiography. A dramatic improvement in hardware of video camera and motion analysis in the recent years allows frequency analysis of motions of animals, including free-swimming zebrafish [18]. The fish heart comprises a single atrium and a single ventricle, which facilitates optical measurement or video imaging analysis of heartbeat in immobilized embryos and larvae [19–22]. On the other hand, heart movement in adults of zebrafish and medaka was analysed using infrared light illumination or high-frequency ultrasound, but these methods still require immobilizing adult fish with anesthetics and special devices [12, 23].

Zebrafish and medaka have recently been established as model animals of human diseases [24–27]. Several pharmacological studies have revealed that larval-stage zebrafish express receptors for sympathetic and parasympathetic neural transmission are expressed in zebrafish larvae and that heart rates in zebrafish larvae change in response to both sympathetic and parasympathetic input with or without anesthesia [17, 21, 28–30], although one of these studies further demonstrated that the autonomic components of the reflex are poorly developed in 5-days-old larvae of zebrafish, suggesting that ANS function is incompletely developed at this stage [21]. Moreover, to evaluate long-term changes in ANS activity such as those induced by environmental changes, it is necessary to study adult animals to avoid major alterations in ANS activity during embryonic development, growth or sexual maturation. Several pioneering studies of the ANS functions have been conducted in adult zebrafish and development of transparent zebrafish lines is promoting the physiological study of heart rate in adult zebrafish [31–33].

Medaka is a small teleost that is native to East Asian freshwater systems and has been another popular laboratory fish for human diseases study, because they can be easily maintained in laboratories, their genetics and development have been revealed in detail, and their whole genome has been sequenced [34, 35]. In addition, medaka have several characteristic features that facilitates the physiological study of ANS activity in unanesthetized adults: they are highly adaptive to a wide range of temperatures as well as to low oxygen content, prefer slowly flowing water and do not swim vigorously. The transparent strain, SukeSuke (SK2), has been established by crossing several spontaneous body-coloring recessive mutants [36, 37] and the heart movement of adult SK2 medaka can be observed through their transparent peritoneum. The present study determines the frequency characteristics of HRV modulation by the ANS and non-invasively quantifies ANS activities by spectral analyses of HRV in unanesthetized medaka using high-speed movie images of the heart movement.

## Materials and methods

### Medaka strains and husbandry

Medaka (*Oryzias latipes*) strain SukeSuke (SK2) reared in laboratory tanks was obtained from our breeding colony. All experiments were conducted with adult fish over 3 months-old and whose body lengths were 2.5 ± 0.2 cm (n = 25 in total). We also analyzed embryos at 6 days post-fertilization at the stage when the heart becomes functional (embryonic stage (St.) 36, n = 5) [38]. The SK2 strain is homozygous for three recessive pigmentation mutations (*b*^*g8*^; null melanophore, *lf*; leucophore free, *gu*; guanineless) [36, 37] and without apparent abnormalities in cardiac activity during embryogenesis and at adult. The fish and embryos were maintained under standard laboratory conditions at 26°C with a 14:10-h light-dark cycle in an incubator at 26°C. All experiments were performed between 15:00–17:00 to avoid diurnal fluctuation and two fish were used for experiments per day. In the experiment to investigate the effects of continuous lighting, we maintained 6 fish in tanks under light for 24 h/day for one week before HRV measurements. Committees for Institutional Animal Care of the Japan Aerospace Exploration Agency and of the University of Tokyo approved the animal protocols.

### Pharmacology and reagents

Atropine (1 mM), propranolol (100 μM) or MS-222 (80 μg/mL Tricaine, Sigma-Aldrich) were added into a bath in which the fish was kept in a smaller observation container. The concentrations of the chemicals used here were about 10 times higher than the previous experiments and we inspect visually the heart rate changes since penetration of the chemicals into the adult fish was expected low [15, 39]. Fish were acclimated to the observation container for 5 min before the administration of atropine or propranolol. and single fish was treated with the drug at a time per video acquisition. Observation was continued while the drug was administered, and a video image was captured after bradycardia or tachycardia was induced. MS-222 was administered for a minimum of 5 min before assays. Images of the heart area were taken for 20 min throughout the measurements.

### Imaging system and digital video recording

Digital video recording of cardiac activities of adult fish and juveniles were conducted as described previously (S1 Fig) [40]. Water and oxygen and maintained at 25 ± 1°C throughout the measurement. The swimming area for the fish of the observation container was restricted but without affecting ventilation and the heart rate of the fish in the container was not altered for any longer than two hours without the chemical administration. Each measurement was completed within 20 min, including the time to place the embryos into container and to establish a quiet, resting and steady state (usually within about 5 min). Heart movement in adult medaka was recorded by taking videos of the ventral view of adult SK2 through the transparent peritoneum using an inverted stereomicroscope (LEICA Fluorescent Dissecting Microscope MZFLIII with a PLANAPO 1.0X lens) equipped with a digital high-speed camera (CASIO Exilim EX-F1) at 2.0X magnification (S2 and S3 Figs) [41]. Single fish was used at once for each image acquisition.

Dechorionated embryos at St. 36 [39] were immobilized in a suitable orientation for video recording using 2.5% methylcellulose in glass-bottomed dishes (Matsunami, Osaka, Japan) and then heart movement was recorded using a stereomicroscope equipped with a digital high-speed camera (EX-F1) as described before (S2 and S3 Figs) [42]: digital video at 300 frames per second (fps) with a resolution of 512 x 384 pixels were captured for up to 20 min and recorded in a PC using Final Cut Pro software (Apple Computer). Since 2–5 min data acquisition is recommended for HRV analysis in humans [4] and heart rate is faster in medaka than in humans, we acquired the data for 3 minutes after the recovery of heart rate which increased due to the move into the observation container. We defined a stable heart rate as steady-state, which was almost same (± 10 bpm) in the first and the last 3 min.

### Extraction of cardiac activity

We extracted the cardiac activities of adult fish, juveniles, larvae and embryos as described previously (S3 and S4 Figs, S1 File) [40, 42]. Four boxes of the same shape were placed in parallel, of 3 boxes included the heart area and one box did not include the heart area which was for the subtraction of the body movement. Ideally the boxes were horizontally aligned to the movement of the heartbeat. The pixel intensities of each ROI were digitalized throughout the entire time series examined using Bohboh software (Bohboh Soft, Tokyo, Japan) and further processed using Cutwin mathematical software (EverGreen Soft, Tokyo, Japan). The average of the three boxes placed on the heart area was calculated to be time-to-time intensity of heart with body motion. The difference of the pixel intensity per frame was calculated for the two waveforms: the intensity changes of the heart with body movement and the waveform of the body movement. Then, the heart movement was extracted by the subtraction of the intensity of body from the heart area at the same time point. The slope of the heart wave is extracted by smoothing the slope corresponding to the change by the 21 points before and after the change, and the wave of the heart motion is reproduced by noise removing.

In embryos, using Cutwin software, pixel intensity of the ROIs in the heart images of the immobilized embryos was digitalized, movement-averaged over 21 frames and then local maxima and minima were determined as described above.

### Steady state heart rate, respiratory rate and power spectral analysis of HRV

The period between pixel intensity minima (representing the end of diastole and the beginning of the contraction at the systole) provided the interbeat interval, from which we calculated beat-by-beat heart rate. We then averaged beat-by-beat heart rates during collection for 3 min to generate steady-state heart rates. Respiratory rates per minute were determined by counting the number of opercula movements in 30 sec of data collection for 3 min. Beat-by-beat heart rates were linearly interpolated and resampled at 8 Hz to create an equidistant time series for spectral analyses of HRV. The time series of heart rates was initially detrended with third-order polynomial fitting and then subdivided into 512-point segments with a 50% overlap, resulting in five data segments collected over a period of 3 min. Fast Fourier transform (FFT) was applied to obtain a power spectrum of HRV on each Hanning-windowed data segment and subsequently the power spectra of the five segments were subsequently averaged to calculate the autospectrum of HRV acquired during the 3 min. The minimal resolution of these spectra was 0.015625 Hz. The data processing described above were conducted using DADiSP software (DSP Development, Cambridge, MA, USA).

### Statistical analyses

Data were statistically evaluated using a one-way ANOVA followed by a comparison with control (Dunnett’s post hoc test) using JMP software (SAS Japan, Tokyo, Japan). A P value of ≤ 0.05 was considered statistically significant. Data are presented as means ± s.d. (standard deviations) of five fish or embryos per experiment.

## Results

### Steady-state heart rate and respiratory rate

**Fig 1:**
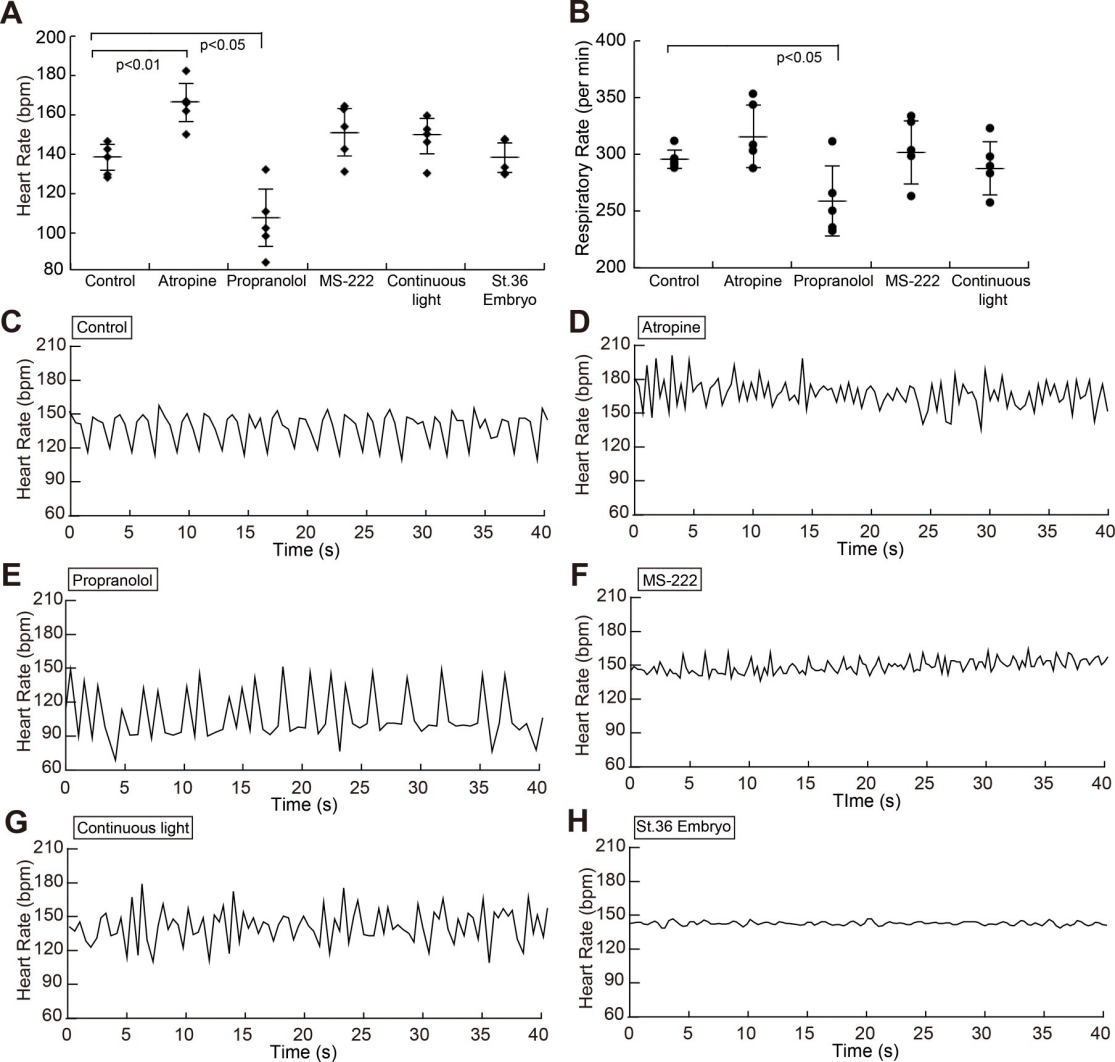
Average of steady-state heart rates, respiratory rates and examples of beat-by-beat heart rates. Group averaged steady-state heart rates were plotted under each condition (A). Group averaged respiratory rates (per min) were plotted under each condition in adult medaka (B). Standard deviations among five fish are plotted as error bars. Examples of beat-by-beat heart rate changes for 40 s under each experimental condition are presented (C-H). Control (C), 1 mM atropine (D), 100 μM propranolol (E), anesthesia with 80 μg/mL MS-222 (F), continuous light (G) and St. 36 embryo (H).

The steady-state heart rate in the control fish was 137.1 ± 6.70 bpm (Fig 1A, S1 Table, n = 5). Atropine increased the rate to 164.8 ± 9.69 bpm (n = 5, *p* = 0.009), showing that atropine induced tachycardia compared with the control, whereas propranolol induced bradycardia by decreasing the heart rate to 106.3 ± 14.6 bpm (Fig 1A, n = 5, *p* = 0.047). The steady-state heart rates in adult fish under anesthesia with 80 μg/mL of MS-222 and under continuous light conditions, were 149.6 ± 12.0 (n = 5) and 147.9 ± 9.07 (n = 5) bpm, respectively (Fig 1A), which did not significantly differ from those of the control (*p* = 0.20 and 0.37, respectively).

The respiratory rates in the control fish was 296 ± 8.22 per min (Fig 1B) and in adult fish administered with propranolol were 259 ± 31.1 per min (Fig 1B), and significantly decrease from control values (*p* = 0.033). The respiratory rates in adult fish administered with atropine and MS-222 and adult fish under continuous light were 316 ± 27.7, 302 ± 28.0 and 288 ± 23.6 per min, respectively (Fig 1B), which did not significantly differ from control values.

### Control HRV

**Fig 2:**
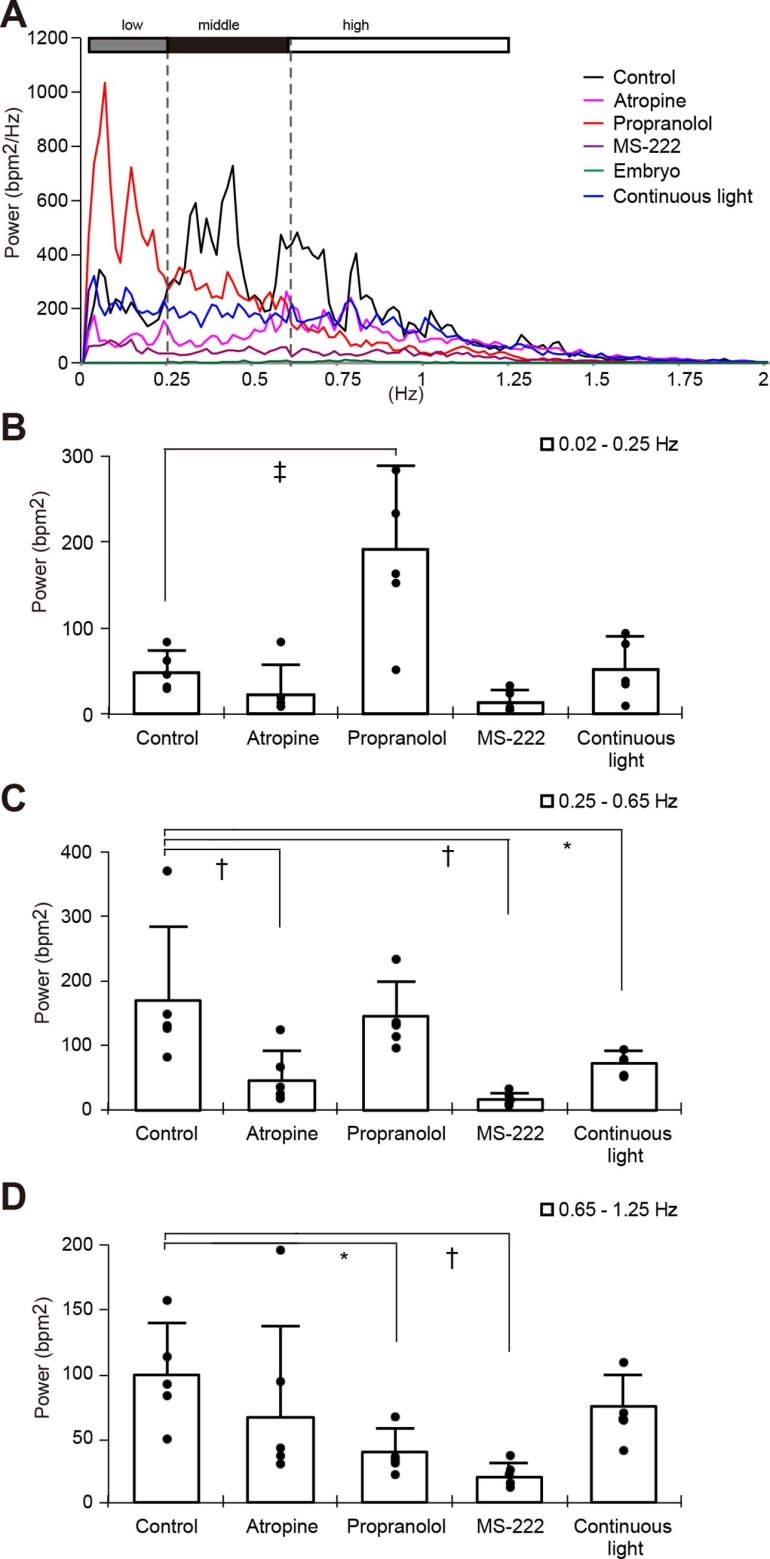
Power spectral analysis of HRV for 3 min in medaka. Power spectral density of HRV in adult control fish, administered with 1 mM atropine, 100 μM propranolol, 80 μg/mL MS-222 and under 1-week continuous light and St. 36 embryos were obtained by fast Fourier transformation using DADiSP software. The mean of power spectral density of HRV in five fish under each condition is shown. Low (0.015625–0.25 Hz), middle (0.25–0.65 Hz) and high (0.65–1.25 Hz) frequencies are shown as gray, black and white bars, respectively (A). The amount of power was added in each frequency range and error bars are standard deviations among five fish. Data from individual fish are indicated as black dots (B–D). B, 0.01625–0.25 Hz. C, 0.25–0.65 Hz. D, 0.65–1.25 Hz. *P ≤ 0.05, ^†^*P* ≤ 0.01, ^‡^*P* ≤ 0.005, compared with control.

Fig 1C shows an example of heart rate over a 40-sec period within a 3-min sample from a control fish. Specific rhythms seemed to emerge in the form of definite heart rate fluctuations. The mean of the power spectral density of the HRV in five adult intact fish (control) shows oscillatory periods at frequencies below 1.25 Hz, and at least two specific peaks (Fig 2A, S2 Table, black line). Power spectral density at a frequency of 0 Hz was omitted from this analysis and the power spectrum was divided into low- (0.02–0.25 Hz, Fig 2B), middle- (0.25–0.65 Hz, Fig 2C) and high- (0.65–1.25 Hz, Fig 2D) frequency ranges. The power of these ranges in the control fish was 48.2 ± 24.8, 171 ± 115 and 98.9 ± 40.3 bpm^2^, respectively.

### Effect of inhibitors for autonomic nervous system on HRV

Fig 1D–1F show the heart rate fluctuation induced by 1 mM atropine, 100 μM propranolol and 80 μg/mL of MS-222, respectively. The mean of the power of the HRV in five adult fish that were administered with atropine, propranolol and MS-222 of each was shown in Fig 2A (n = 5). The mean power of the low-, middle- and high-frequency ranges in the presence of atropine was 22.8 ± 34.3, 48.0 ± 44.9 and 66.2 ± 70.9 bpm^2^, respectively. The power of the low-, middle- and high-frequency ranges with propranolol administration was 191 ± 96.2, 147 ± 54.4 and 39.4 ± 17.8 bpm^2^, respectively. And those in the low-, middle- and high-frequency ranges under MS-222 anesthesia was 13.9 ±13.8, 18.0 ± 9.40 and 20.3 ± 10.1 bpm^2^, respectively. Atropine significantly reduced the power in the middle-frequency range but propranolol did not (Fig 2C). On the other hand, propranolol increased and decreased the power in the low- and high-frequency ranges while atropine had not effect on variability in these ranges (Fig 2B and 2D, respectively). Reductions in the powers of the middle- and high- frequency ranges were statistically significant in MS-222 administered fish (Fig 2B– 2D).

### HRV under continuous light

Continuous light stimulation was expected to disturb ANS modulation. Therefore, we analyzed the heart rate fluctuation in adults after one week under continuous lighting (Fig 1G). The power of the low-, middle- and high-frequency ranges of these fish was 51.7 ± 38.2, 75.2 ± 18.7 and 75.0 ± 24.6 bpm^2^, respectively (Fig 2A blue line; Fig 2B– 2D). The power in the middle-frequency band was significantly reduced under continuous light for 1 week (Fig 2C).

### Development of ANS modulation in medaka

**Fig 3:**
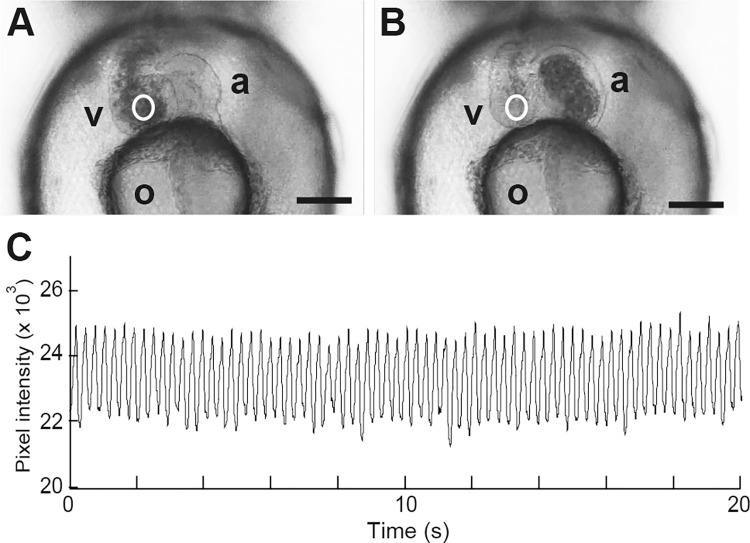
Automated extraction of heart movement in embryos. Sample pictures are at diastole and systole (A and B) in embryonic heart area view. Pixel intensities in the white circular ROI were extracted as numerical data. Twenty seconds of total intensity in white circles throughout the entire series were plotted after being smoothed by the 21-point moving average (C). a, atrium; v, ventricle. o, oil droplets. Scale bars indicated 100 μm.

Fig 3A and 3B show the typical picture of diastole and of systole, respectively. Fig 3C shows changes in intensity inside the white ROI in the heart area as an indicator of heartbeat for 20 sec (Fig 3A and 3B; white circles). Thus, heartbeats were clearly determined from changes in pixel intensity of the heart area in St. 36 medaka embryos.

**Fig 4:**
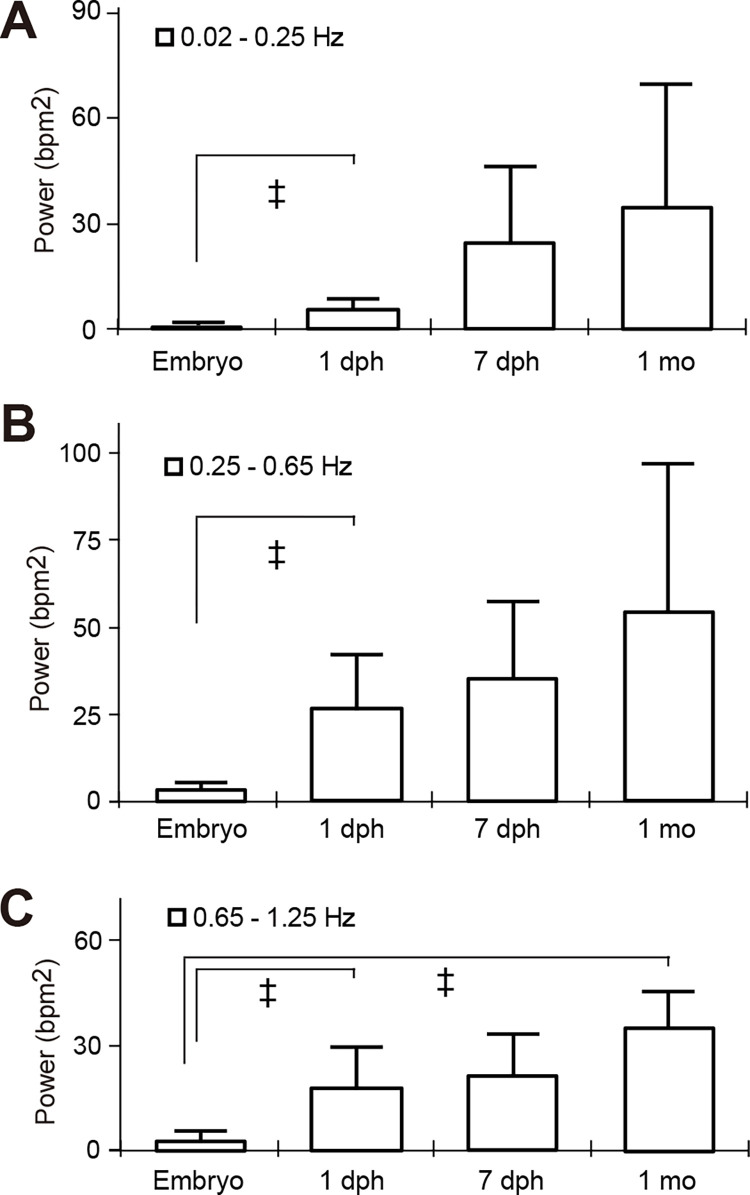
Power spectral analysis of HRV for 3 min during development. Power spectral density and the mean of power spectral density of HRV in st.36 embryos (n = 5), 1 day post hatch (dph) larvae (n = 20), 7 dph larvae (n = 3) and 1 month old juvenile (n = 4) was calculated by fast Fourier transformation using DADiSP software. Low (0.015625–0.25 Hz), middle (0.25–0.65 Hz) and high (0.65–1.25 Hz) frequencies are shown in panels A, B and C, respectively. The amount of power was added in each frequency range and error bars are standard deviations among samples. A, 0.01625–0.25 Hz. B, 0.25–0.65 Hz. C, 0.65–1.25 Hz. ^‡^*P* ≤ 0.005; compared with st.36 embryos.

The beat-by-beat heart rate in St. 36 embryos essentially remained consistent with minimal fluctuation (Fig 1H). To evaluate the development of the ANS modulation during fish growth, we took the high-speed videos of heart movements and analyzed the HRV in st. 36 embryos (n = 5), 1 day post hatch (dph, n = 20) and 7 dph larvae (n = 3) and 1 month old juvenile medaka (n = 4). The mean power of the low frequency ranges (0.02–0.25 Hz) in embryos, 1 dph, 7 dph and 1 month old medaka was 0.32 ± 0.31, 5.43 ± 2.91 (*p* < 0.001), 24.4 ± 21.7 (*p* = 0.101) and 34.6 ± 34.9 (*p* = 0.104) bpm^2^, respectively (Fig 4A, S3 Table). The mean power of the middle frequency ranges (0.25–0.65 Hz) in each stage fish was 3.39 ± 2.11, 26.3 ± 15.7 (*p* < 0.001), 35.0 ± 22.0 (*p* = 0.063) and 53.7 ± 41.0 (*p* = 0.677) bpm^2^, respectively (Fig 4B). The mean power of the high frequency ranges (0.65–1.25 Hz) in each developmental stage was 3.05 ± 2.08, 18.0 ± 11.8 (*p* < 0.001), 21.2 ± 11.6 (*p* = 0.054) and 35.3 ± 10.8 (*p* = 0.006) bpm^2^, respectively (Fig 4C). There was a tendency of increase in the ANS modulation in all frequency ranges as the fish grew.

## Discussion

We measured steady-state heart rate and HRV in intact adult medaka by extracting heart movements from ventral high-speed video images and spectral analysis. We then defined the characteristics of the HRV modulation by the parasympathetic and sympathetic nervous systems in adult medaka based on the following findings.

### Steady-state heart rate measurement and HRV analysis in medaka

Previous studies of steady-state heart rates in adult medaka have mainly focused on the effects of temperature on the heartbeat of the isolated heart or on the heart of intact adult medaka. These studies found that the steady-state heart rate of medaka is about 140 bpm at 25°C with or without anesthesia [43–48], with which our findings are consistent. Despite the interest in comparative studies of cardiac regulation in vertebrates, only a few investigators have applied spectral analysis to non-mammalian vertebrates [49, 50]. The wide diversity of cardiac-related signals, non-standardized procedures and techniques might have hindered the application of spectral analysis to fish [10]. Although several studies have examined the involvement of ANS activity in heart rate regulation, variations in specific frequency ranges of the HRV in fish have not been quantified.

We found here that specific peaks appear in the HRV spectrum of adult unanesthetized medaka and that the power spectrum of HRV in these fish covers at least three frequency ranges (0.02–0.25, 0.25–0.65 and 0.65–1.25 Hz) presumably because of the regulatory machineries discussed below.

### Contribution of the ANS to HRV

Atropine, a muscarinic receptor antagonist, reduced the fluctuations in the range of 0.25–0.65 Hz (middle-frequency range) and induced tachycardia. On the other hand, propranolol, a β-adrenergic receptor antagonist, reduced the fluctuation in the range of 0.65–1.25 Hz and induced bradycardia. These results suggested that the parasympathetic and sympathetic nervous system primarily modulate HRV in the middle- and high-frequency ranges, respectively. Anesthesia with MS-222 suppressed the fluctuations within both the 0.65–1.25 Hz and 0.25–0.65 Hz ranges, strengthening the finding that anesthetics block both the sympathetic and parasympathetic nervous systems [51].

Such correspondence of the sympathetic or parasympathetic nervous system with two frequency bands in medaka seems to contradict to the mammalian system. It is considered that the central circuit in the mammalian sympathetic nervous system works slowly because of the network complexity which is due to the increased number of synapses as a result of the evolutionary process [52, 53]. As well as mammals, teleost fish have a true ganglionated sympathetic trunk and a distinct vagal system, strongly suggesting that these fish have a simpler sympathetic circuit than that of mammals. In addition, respiration is slower than the heart rate in mammals, in which the high frequency range of HRV refers to vagal nerve activity and mainly reflects respiratory sinus arrhythmia. In contrast, ventilation caused by opercular movement is faster than the heart rate in fish and the reported frequency range of opercular movement in medaka is 4–5 Hz at 25°C [43]. Therefore, the effect of respiratory sinus arrhythmia seen in mammals on HRV would be quite small in teleost fish. Evolutionary aspects and respiration style might explain the difference in autonomic modulation of the frequency bands between medaka and mammals.

Although both atropine and anesthesia by MS-222 tended to suppress fluctuations in the low-frequency range, the difference did not reach statistical significance and the fluctuation in this range considerably differed among individual adult fish. Moreover, propranolol remarkably increased the fluctuations in the low-frequency range: the propranolol-administered adult medaka occasionally showed rapid movement in addition to bradycardia, presumably because blood flow was decreased and the power in the low frequency range increased. Such increased power could also be attributable to the ventricular arrhythmia caused by the bradycardia and whether the power in the low-frequency range induced in this manner can be useful as an indicator of the ANS activities requires further consideration. It would be helpful to examine HRV especially at this frequency range using electrocardiography.

The finding that both atropine and propranolol affected spontaneous HR in awake and spontaneously breathing medaka, means that there were ongoing sympathetic and parasympathetic drives to the pacemaker in the fish, originating either from central autonomic centers or the intracardiac nervous system, or both. It is usually considered that at any given time either sympathetic or parasympathetic influences predominate on the pacemaker, but this view might be simplistic and the current finding that both inputs were active at once indicates that the ANS control of the heart is complex also in teleost fish.

### Influence of environmental disturbance on cardiac ANS

We evaluated the effects of an environmental disturbance, which was caused by exposure to constant light for one week, on the ANS activity in adult medaka and found that the HRV decreased only in the middle-frequency range. Since fluctuations in the middle- and high-frequency ranges could indicate parasympathetic and sympathetic nervous activity, respectively, the data suggest that only parasympathetic nervous activity was reduced under continuous light in adult medaka, whereas sympathetic nervous activity was less affected.

### Development of the ANS modulation in medaka

HRV was rarely observed in st.36 medaka embryos before hatching and HRV tended to increase during one month growth after hatching, although it is reported that cardiac branches of the autonomic nerve have developed in medaka embryos before hatching [42]. These results suggest that it might take at least one month for the ANS to be fully functional after nervous fibers of ANS innervate the heart.

### Advantages of using adult medaka as a model animal

Our findings demonstrated that HRV is regulated by both the sympathetic and parasympathetic nervous systems and that environmental stimuli and anesthesia can both alter the power spectrum of the HRV in adult medaka. We also found that heart rate is consistent in medaka embryos and that the ANS modulation of HRV has not developed in St. 36 embryos of medaka, unlike in zebrafish. The HRV measurement by high-speed video imaging and spectral analysis in unanesthetized adult medaka might offer advantages to investigate the impacts of the environmental stress on the ANS activities in vertebrates.

Small teleost like medaka can be reared under the same experimental conditions from egg to adult and experimental conditions including breeding temperature, lighting schedule and feeding conditions can be easily controlled. Although small teleost are difficult to manipulate and there are still difficulties to evaluate cardiac activities, small teleost have advantageous for studying the effects of environmental conditions on vertebrate ANS activity and its development. The medaka system also confers advantages for drug screening and phenotypic analysis of spontaneous or genetically manipulated mutants to develop drugs that can control the functions of the autonomic nervous system.

A type II error in the present study is possible due to the low sample size. Although HRV in the 0.25–0.65 Hz range decreased significantly in the anesthetized adult medaka and in those under constant light conditions, the steady-state heart rate did not differ from the controls despite the tendencies to tachycardia, suggesting that the HRV spectrum can be a more sensitive index to detect different modulation by ANS than the steady-state heart rate. It is also possible that the resampling process acted as a low-pass filter, causing attenuation in the high-frequency range.

The opercula movements tended to increase and decrease under the administration of atropine and propranolol, respectively, but there were no significant differences. Since fish exchange gases through branchial respiration and more energy is required for ventilation with gills than with pulmonary respiration due to the low oxygen content and high density of water as a respiratory medium [54], HRV might be regulated by respiratory rate differently in fish than in mammals, despite the correlation between heart rate and respiratory rate [55, 56]. Since we excluded the effects of ventilation movements by data processing before spectral analysis and the number of the opercular movements did not significantly differ, the influence of ventilation frequency in HRV might be successfully minimized in the current study.

In summary, we identified the steady-state heart rate in medaka by extracting heart movement from ventral video images, and characterized a part of the frequency nature of HRV modulation with spectral analysis in adult not-anesthetized medaka. Atropine significantly reduced HRV in the middle-frequency range, suggesting primarily parasympathetic nervous regulation of HRV within this range. On the other hand, propranolol reduced HRV in the high-frequency range, suggesting sympathetic nervous regulation within this frequency range. HRV was not observed in pre-hatching embryo and tended to increase during one month growth after hatching. Moreover, constant light reduced HRV only at the middle-frequency range, suggesting that parasympathetic nervous activity was suppressed. These findings will contribute to the understanding of the maturation process of the ANS in vertebrates and the precise modulation of the ANS by environmental stimuli.

## Supporting information

S1 Fig Trough for video recording. A: Trough for fry. One day old fry was put into the arrow area. B: Trough for adult fish. Holes were made on the front and the back (on the left and right in this picture, respectively) wall of the trough for ventilation.
(JPG)

S2 Fig The method for extracting the heart movement. 1 and 2. The movie was acquired through the inverted dissection microscopy. Movie was taken around 20 min with 300 fps. The 3 min image series in which the fish stayed at the same position was extracted from the 20 min movie. 3. Heart rate analysis.
(JPG)

S3 Fig Settings of the video recording and pictorial examples. A: Down light, B: Sidelight, C: Sample stage, D: Inverted stereomicroscope (Leica MZ FLIII), E: Detection camera (CASIO EXLIM F1), F: 1 week old fry, G: Adult medaka. Ey; eye, H; heart, O; oil droplet. Scale bars indicated 100 μm.
(JPG)

S4 Fig Example plot of the extraction of cardiac movement. Blue boxes in the left panels indicate the pickup areas including the heart and green boxes indicate the areas to pick up body movement. Blue and green lines indicate pixel intensity of the blue box on the heart area and the green box outside of the heart, respectively. Black line indicates the subtracted intensity of green from the blue line.
(JPG)

S1 Table Original data for Fig 1. Beat by beat heart rate and counted respiratory rate.
(XLSX)

S2 Table Original data for Fig 2. Actual FFT power spectrum data of each experimental condition.
(XLSX)

S3 Table Original data for Fig 2. Actual FFT power spectrum data of each developmental stage.
(XLSX)

S1 File Image analysis. Applications and formula used in this study.
(DOCX)
