# Supplementary figures and images for: Acquirement of the autonomic nervous system modulation evaluated by heart rate variability in medaka (Oryzias latipes)
Source: PLoS One. 2022 Dec 30;17(12):e0273064. doi: 10.1371/journal.pone.0273064 (PMC9803310; doi:10.1371/journal.pone.0273064)

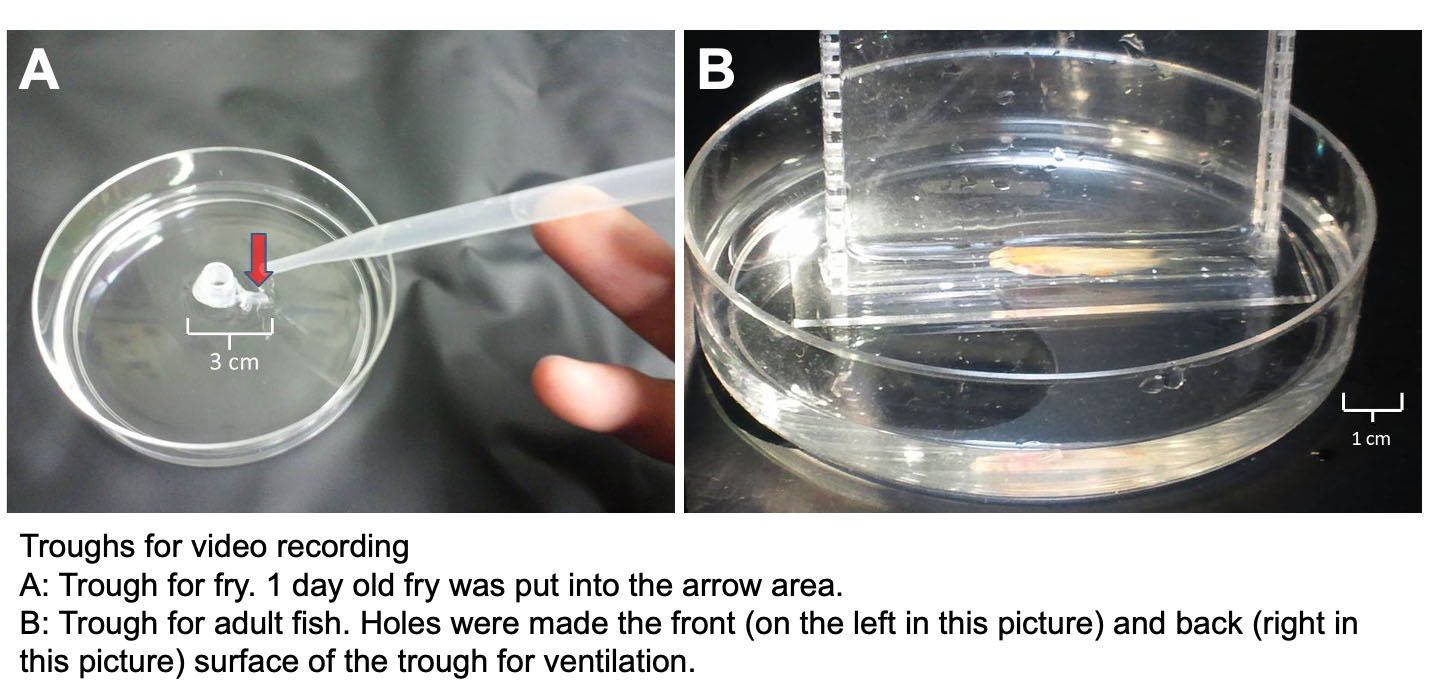

Supplement: S1 Fig — A: Trough for fry. One day old fry was put into the arrow area. B: Trough for adult fish. Holes were made on the front and the back (on the left and right in this picture, respectively) wall of the trough for ventilation. (JPG) [file pone.0273064.s001.jpg]

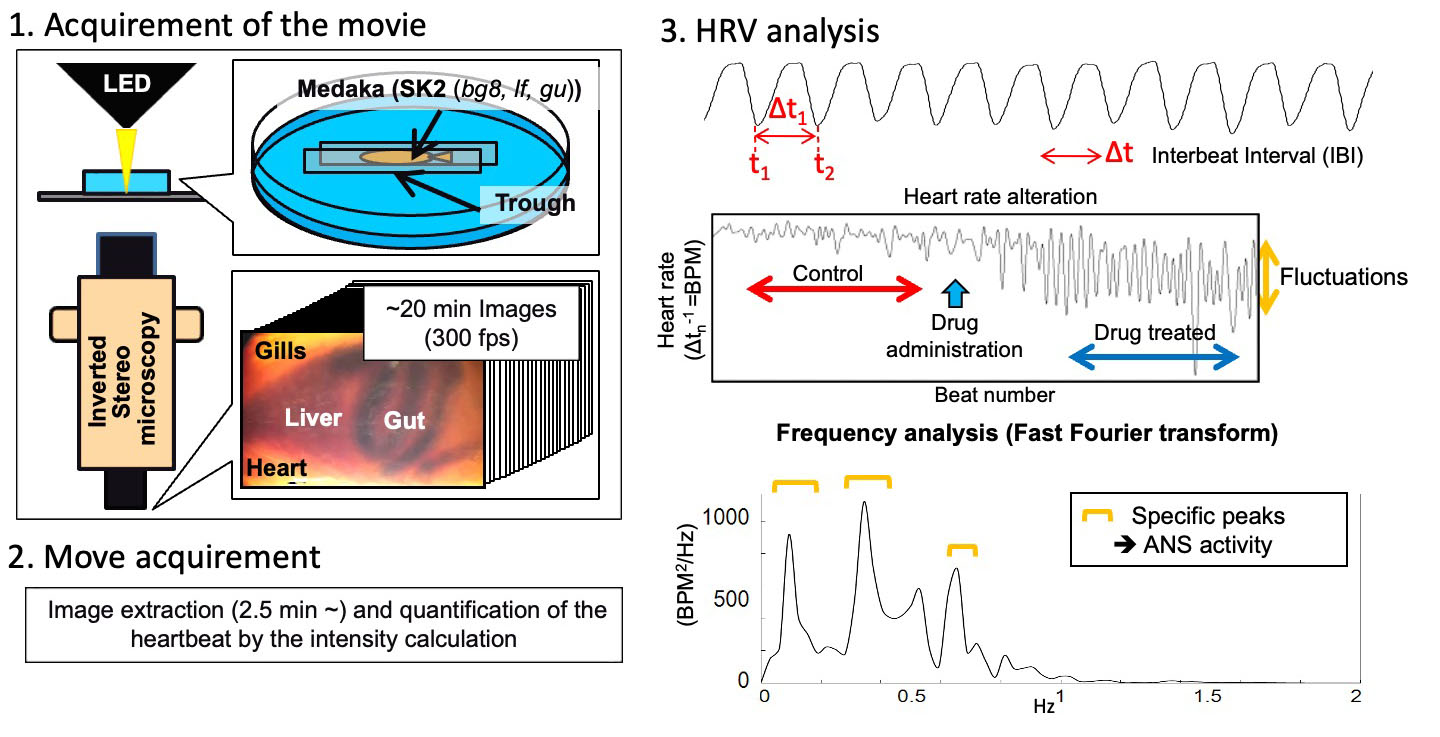

Supplement: S2 Fig — 1 and 2. The movie was acquired through the inverted dissection microscopy. Movie was taken around 20 min with 300 fps. The 3 min image series in which the fish stayed at the same position was extracted from the 20 min movie. 3. Heart rate analysis. (JPG) [file pone.0273064.s002.jpg]

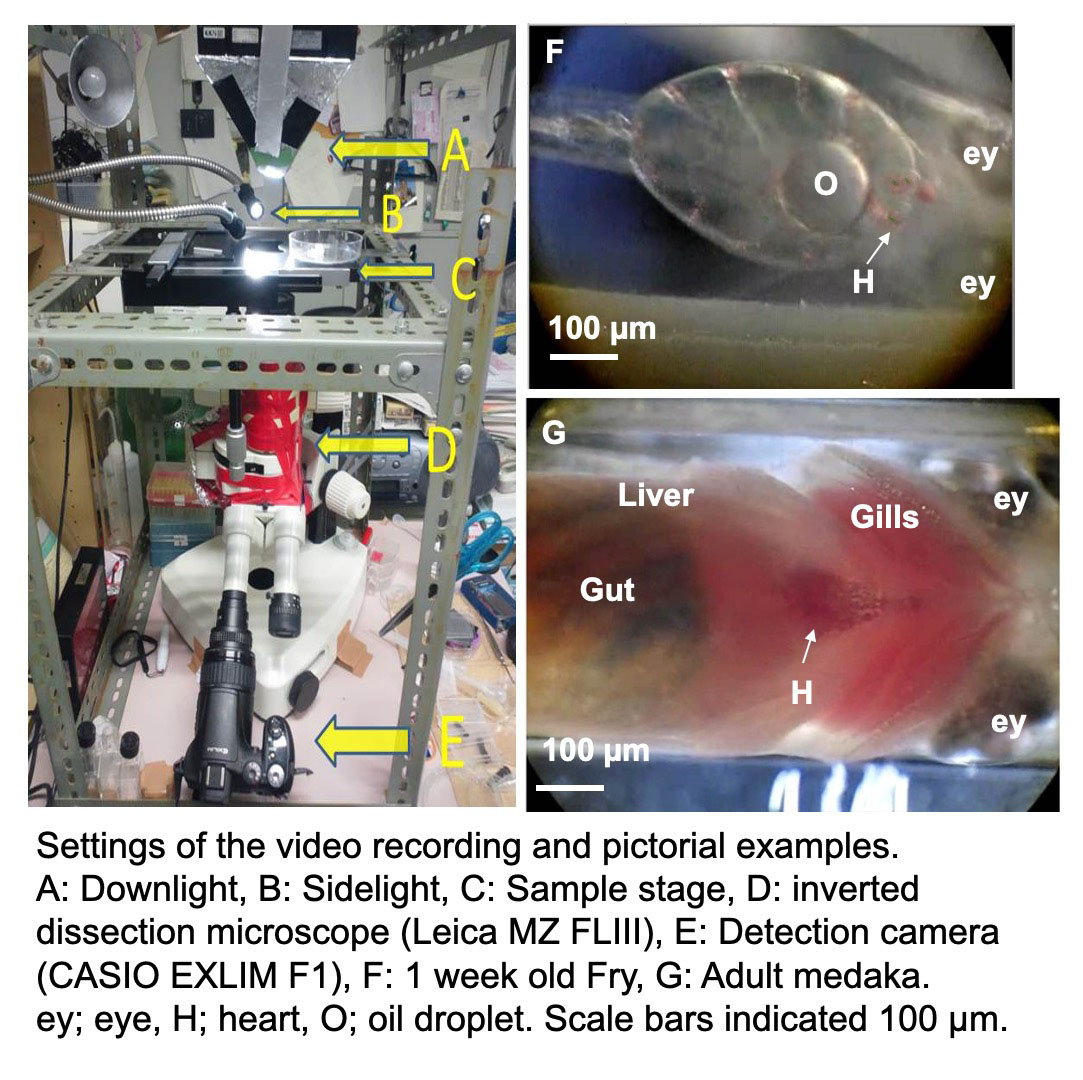

Supplement: S3 Fig — A: Down light, B: Sidelight, C: Sample stage, D: Inverted stereomicroscope (Leica MZ FLIII), E: Detection camera (CASIO EXLIM F1), F: 1 week old fry, G: Adult medaka. Ey; eye, H; heart, O; oil droplet. Scale bars indicated 100 μm. (JPG) [file pone.0273064.s003.jpg]

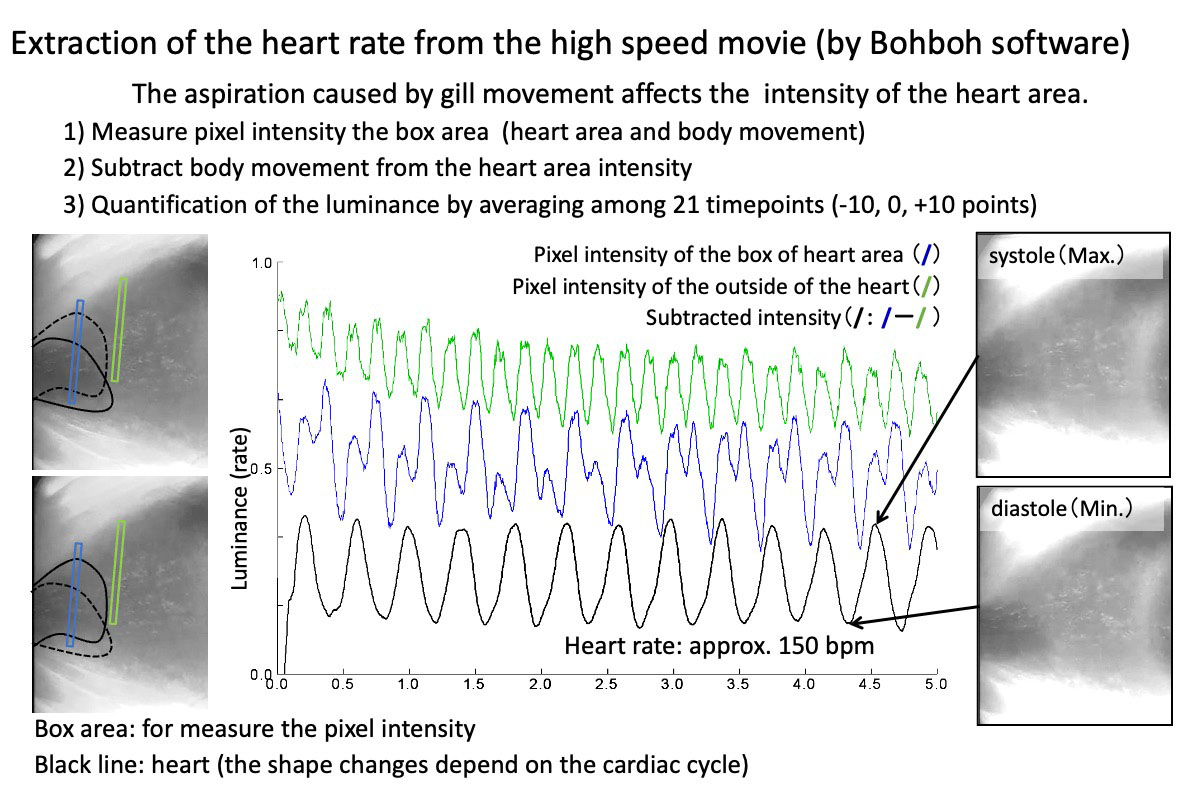

Supplement: S4 Fig — Blue boxes in the left panels indicate the pickup areas including the heart and green boxes indicate the areas to pick up body movement. Blue and green lines indicate pixel intensity of the blue box on the heart area and the green box outside of the heart, respectively. Black line indicates the subtracted intensity of green from the blue line. (JPG) [file pone.0273064.s004.jpg]
