# Supplementary material for: Acquirement of the autonomic nervous system modulation evaluated by heart rate variability in medaka (Oryzias latipes)
Source: PLoS One. 2022 Dec 30;17(12):e0273064. doi: 10.1371/journal.pone.0273064 (PMC9803310; doi:10.1371/journal.pone.0273064)
Supplement: S1 File — Applications and formula used in this study. (DOCX) [file pone.0273064.s008.docx]

Bohboh:

Place 4 boxes of the same shape in parallel, of 3 boxes includes the heart area and one box not to be included the heart, which is for the subtraction of the body movement. Ideal box locations are horizontally aligned to the movement of the heartbeat. However, the most important point is the subtracted box should not include the heart area but close to it. Then, calculate the pixel intensity of each box in whole time series.

Cutwin:

Take the average of the three Boxes ([2][@], [4] [@], [6] [@]) placed on the heart to create a time to time intensity of heart with body motion.

loop(1,num([2]),1){[9][@]=([2][@]+[4][@]+[6][@])/3}

The difference of the pixel intensity per frame is calculated for the two waveforms: the intensity change of the heart with body movement ([10][@])and the waveform of the body movement ([11][@]). Then, the heart movement ([12][@]) is extracted from the subtraction of the intensity of body from the heart area at the same time point. The slope of the heart wave is extracted by smoothing the slope corresponding to the change by the 21 points before and after the change, and the wave of the heart motion is reproduced by removing noise ([13][@]).

loop(2,num([9]),1){[10][@]=[9][@]-[9][@-1]}

loop(2,num([8]),1){[11][@]=[8][@]-[8][@-1]}

loop(2,num([10]),1){[12][@]=[10][@]-A*[11][@]}

smooth1(21,[12],[13])

The slope values are smoothed again to derive a larger trend.

smooth1(71,[13],[18])

From the slope that has been ascertained as a rough trend, the point at which the slope turns from negative to positive, i.e., the minimum, which means the point at the contraction began, is extracted and the number of that point at the same time.

[19][1]=0

loop(1,num([18])-1,1) {if([18][@]<0){if([18][@+1]>0){if(max([19])<1){[19][@]=1}else{[19][@]=max([19])+1}}}}

loop(1,max([19]),1){[20][@]=peak_cell([1],[19],1,@)}

Remove the noise.

loop(1,num([20]),1){[21][@]=[20][@]-35;if([21][@]<0){[21][@]=1}}

loop(1,num([20]),1){[22][@]=[20][@]+35;if([22][@]>num([13])){[22][@]=num([13])}}

loop(num([21]),2,-1){if([21][@]<[22][@-1]){[21][@]=[21][@-1];cell_del([20],@);cell_del([21],@);cell_del([22],@)}}

Calculate the duration of each peak. Convert to BPM

loop(1,num([20]),1){#1=[21][@];#2=[22][@];#3=@;loop(#1,#2,1){[23][@-#1+1]=[14][@];[24][#3]=min_cell([23])+#1-1}}

loop(2,num([24]),1){[25][@]=[24][@]-[24][@-1]}

loop(num([25]),1,-1){if([25][@]>=0){cell_del([24],@);cell_del([25],@)}}

loop(2,num([25]),1){[26][@]=18000/[25][@]}

cell_del([26],1)

loop(2,num([25]),1){[27][@-1]=[24][@]-[24][2]+1}

Frequency analysis using the data [26] and [27].
